# Aging characteristics of drip irrigation belt in Xinjiang cotton fields and their effects on its recovery and recycling

**DOI:** 10.1038/s41598-023-43094-x

**Published:** 2023-10-07

**Authors:** Junhui Ran, Yu Ren, Wensong Guo, Can Hu, Xufeng Wang

**Affiliations:** 1 College of Mechanical and Electrical Engineering, Tarim University, Alar, 843300 China; 2 Agricultural Engineering Key Laboratory, Universities of Education Department of Xinjiang Uygur Autonomous Region, Tarim University, Alar, 843300 China

**Keywords:** Engineering, Mechanical engineering

## Abstract

The old drip irrigation belts in Xinjiang cotton fields are prone to damage and have low recovering efficiency when being mechanically recovered, as well as poor mechanical performance and short service life of the old materials used in the production of various new products. Therefore, experiments were carried out from the macroscopic mechanical properties and microscopic chemical composition changes of the old drip irrigation belt to explore how the changes in its mechanical and chemical properties affect the recovery and recycling of the old drip irrigation belt. Mechanical properties of the old drip irrigation belts were tested by statics and dynamics test methods. The experiment results of indicators about mechanical properties showed that the tensile strength, elongation at break, natural rebound rate, impact strength and other mechanical parameters of the old drip irrigation belt samples were significantly lower than the corresponding values of the new ones. Which will affect the tensile capacity of the drip irrigation belt when it is recovered in the field. Furthermore, X-ray energy spectrum and infrared spectrum methods were used to analyse the chemical composition changes of the old drip irrigation belt. The determination results of X-ray energy spectrum showed that the oxygen content of the new drip irrigation belt samples was 1.73%, while that of old drip irrigation belt samples reached 12.15% ~ 15.27%. Then, the infrared spectrum experiment results showed that there were significantly more carbon groups in the old drip irrigation belt samples than that in the new ones. In addition, the correlation between the mechanical properties and oxidation degree of drip irrigation belt samples was analyzed, results showed that there was a significant negative correlation between them. This study results can provide basic data and theoretical guidance for the research and development of drip irrigation belt recovery equipment in Xinjiang cotton field, the research of rapid detection method of drip irrigation belt aging, the manufacture of anti-aging drip irrigation belt and the cyclic utilization of old materials.

## Introduction

Xinjiang China, is a typical drought and water shortage area^1^, where water resources account for only about 3% of China's total, the agricultural water is seriously lacking, particularly.The sunshine duration in summer is long, so, the evaporation of surface water is large in Xinjiang^2^. The drip irrigation technology has many advantages, such as significant fertilizer saving, yield increasing, efficient water-saving and labor saving^3^. Therefore, in order to improve agricultural water saving and promote the healthy and sustainable development of Xinjiang's agriculture, the drip irrigation technology has been widely used^4,5^. At present, the area of farmland used drip irrigation technology in Xinjiang has reached about 3.33 × 10^6^ ha. As is an important part of the drip irrigation water-saving system, about 4 × 10^5^ tons of the drip irrigation belts are needed in Xinjiang every year. The single wing labyrinth drip irrigation belt accounts for about 90% of the total of that, mainly used in the cotton field drip irrigation system^6^.

The main component of drip irrigation belt is polyethylene^7^. Which is a polymer material, with service life varies greatly depending on the natural environment^8^. Under the special climatic conditions in Xinjiang, the drip irrigation belt is prone to fracture and large plastic deformation when it is recycled in the field due to its aging during use in the cotton fields. This brings great difficulties to the mechanized recovery of drip irrigation belts approximately 0.12 tons of it from 1 hectare area in the field. The recovered drip irrigation belts can no longer be reused directly^9^. The annual purchase of new drip irrigation belts has increased the cost by 1650 RMB per hectare. Generally, the old drip irrigation belt is cleaned and then crushed into granules. Then, it is mixed with new materials to make new drip irrigation belt or other products^10^. However, because the chemical composition, molecular structure and external mechanical properties of the old drip irrigation belt materials have changed, the products made of the old drip irrigation belt granules often have poor performance in terms of service life and mechanical properties^11^. So, which can not meet the needs of users. Therefore, the old drip irrigation belts have low recycling rates. The old drip irrigation belt has also indirectly caused resource waste and environmental pollution^12^. About 4 × 10^5^ tons of the polyethylene old drip irrigation belts produced annually in Xinjiang have not been recycled with high efficiency and quality. If these old drip irrigation belts are recycled efficiently and scientifically, which will be conducive to cost saving, efficiency enhancement and sustainable development for cotton planting industry. So, it is of great significance to systematically study the aging of drip irrigation belts and its impact mechanism on the mechanical recovery and recycling of drip irrigation belts. This can provide basic data for the design of efficient and high-quality recovery machinery for old drip irrigation belts; and provide scientific guidance for the efficient recycling of old drip irrigation belt materials.

Scholars have carried out some research on the aging performance of polyethylene drip irrigation belt and other polyethylene products under different conditions and the anti-aging effect of different additives on polyethylene materials^13^. Which provides a reference for how to improve the anti-aging performance of polyethylene products to a certain extent. However, there is a lack of comprehensive and systematic research on the aging of drip irrigation belts under specific natural environment. In addition, the adverse effects of aging of drip irrigation belts on mechanized recovery operation, and the specific impact on the cyclic utilization of old drip irrigation belts have not been studied yet. The relationship between anti-aging agent and light aging resistance of PE-LLD was studied. Results showed that the use of specific antioxidants and light stabilizers could improve the anti-aging ability of PE-LLD to some extent^14,15^. Some scholars have also found that PE-LLD can cause cross-linking, breaking and oxidation of molecular chains after ultraviolet irradiation, thus promoting material aging^16^. In addition, the long chain break, lateral group formation, crystal structure change, chain configuration and conformation change caused by aging can be reflected by infrared spectroscopy and X-ray diffraction analysis^17^. Studying the aging behavior of polyethylene in different practical use environments can further explore its aging mechanism and provide reference for its structural design, life prediction and anti-aging agent research^18^. The oxidation induction time method was also used to study the interaction between antioxidants and carbon black in PE materials^19^. Under different experimental conditions such as indoor accelerated aging and outdoor natural exposure aging, PE materials have also been studied on the changes of thermal and mechanical properties before and after aging^16,20,21^. The impact strength, bending strength, tensile strength, elongation at break and so on of PE after outdoor natural aging in specific areas such as Jinan and Lhasa, China, have also been tested^22,23^. Besides, the stress cracking behavior of PE-HD after photooxidation aging was also studied. Experimental results showed that the aging cracking of PE-HD was closely related to the deterioration of the property of the irradiated layer on the material surface and the creep of the samples^24^. Generally, thickened drip irrigation belts are used abroad, and due to the better natural environment compared to Xinjiang, their aging problem is not so prominent and can be used multiple times. Researchers' research on this type of drip irrigation belt mainly focuses on the impact of irrigation parameters on the professional performance of drip irrigation belts, and so on^25^. Therefore, there are currently no research results that can be directly referenced in the aging characteristics of drip irrigation belts and their impact on mechanized recycling, and reuse in Xinjiang cotton fields where low-cost thin drip irrigation belts are used.

It can be seen from the existing research status that under the unique regional climate conditions in Xinjiang, the aging of the old drip irrigation belt in cotton fields and its many impacts on recovery and recycling have not yet been comprehensively studied. Therefore, this article proposed to study the changes in macroscopic mechanical properties and microscopic chemical components of old drip irrigation belts, as well as the correlation between changes in chemical components and mechanical properties, in order to analyze the potential impact of drip irrigation belt aging on its mechanized recovery and recycling. Through this study, it was expected to provide theoretical support and basic data for the research and development of efficient drip belt recovery machinery and the recycling of old materials of drip irrigation belt. Then, the following hypothesises were proposed. Firstly, it was believed that the old drip irrigation belt in Xinjiang region has aged compared to the new drip irrigation belt. Secondly, it was believed that the chemical composition of the old drip irrigation belt has changed after aging, and this change was related to its mechanical properties.

To this end, we conducted relevant research work through the following methods. Firstly, the mechanical characteristics of the old drip irrigation belt were studied experimentally. The indexes of tensile strength^26^, elongation at break^27^, natural rebound rate^28^, plastic deformation rate caused by static load tension and impact strength of the new and old drip irrigation belt samples were tested. Based on the test results of the above mentioned mechanical indexes of different drip irrigation belt samples under static and dynamic force conditions, the change rule of mechanical properties of the old drip irrigation belts can be comprehensively analysed and judged. Then, the aging degree of old drip irrigation belt samples relative to the new ones and its specific impact on the mechanized recovery and cyclic utilization of drip irrigation belt in cotton fields were judged. X-ray photoelectron spectrometer is widely used to detect the types of elements contained in complex substances and their relative content^29^. While the infrared spectrometer is often used to detect the types of chemical groups contained on the surface of substances^30^. So, in this study, in order to better analyze the aging characteristics of drip irrigation belts from the chemical level, X-ray photoelectron spectrometer and infrared spectrometer were used. Then, the differences of chemical elements and functional groups between new and old drip irrigation belt samples were studied. Finally, based on the results of mechanical and chemical component determination experiments of drip irrigation belt samples, the correlation analysis was conducted on the mechanical property parameters of the samples and the chemical components of corresponding samples. Furthermore, the relationships between the aging degree of drip irrigation belt and its mechanical properties and chemical components were judged.

## Materials and methods

### Materials

**Figure 1:**
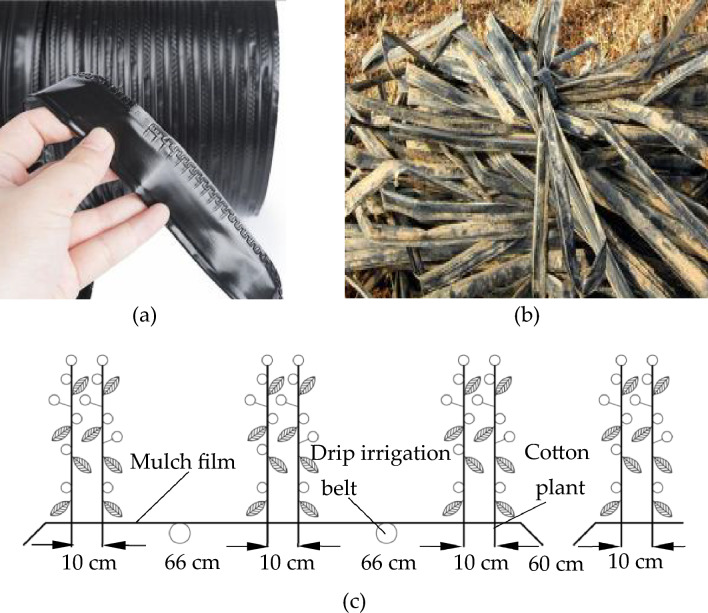
Drip irrigation belts for experiment and its laying mode in cotton field: (**a**) new labyrinth drip irrigation belt; (**b**) old labyrinth drip irrigation belt; (**c**) cotton planting mode.

Because the cotton fields in Xinjiang basically use labyrinth drip irrigation belt (as mentioned in the introduction), therefore, in this study, the labyrinth drip irrigation belt which is widely used in cotton fields was taken as the test material. The labyrinth drip irrigation belt (produced by Xinjiang Hongtianyun Pipe Industry Co., Ltd, Fukang, Xinjiang, China.) used in the experiment has a wall thickness of 0.2 mm and an outer diameter of Φ16 mm, the appearance of new and old labyrinth drip irrigation belts were shown in Fig. 1a,b, respectively. The old drip irrigation belt samples (named as group 1, group 2 and group 3 respectively) were collected in the 3 machine picked cotton fields in Alar, Xinjiang, China. The soil in the fields is all sandy loam soil, and its fertility is basically the same. The other conditions of the cotton field where the old drip irrigation belts were located remained consistent, and some of the main environmental parameters were as follows. The specific cotton planting mode was shown in Fig. 1c; two drip irrigation belts are laid under a single film(with thickness of 0.02 mm), and the distance between the drip irrigation belt and adjacent cotton plants is 33 cm. The time for laying the drip irrigation belt for the experiment was April 20, 2021. The emitter discharge of all experimental cotton fields was 2L/h, and the cotton was irrigated 10 times during the entire growth period, with a cumulative irrigation water volume of 4500m^3^/hm^2^. Additionally, irrigation water comes from the melting snow water of high mountains. During the use of drip irrigation belts, the average temperature under the film in the daytime was about 41.2 °C; the average ultraviolet intensity of sunlight was about 28.9 W/m^2^. Then, the time for recovering the drip irrigation belt after the cotton harvest was October 20. In order to avoid the influence of the drip irrigation belt recovery machinery on the mechanical properties of old drip irrigation belt samples, the samples were manually recovered, then they were thoroughly cleaned for preparation of experiment samples.

### Mechanical characteristics experiment of drip irrigation belt samples

#### Tensile strength experiment of drip irrigation belt samples

**Figure 2:**
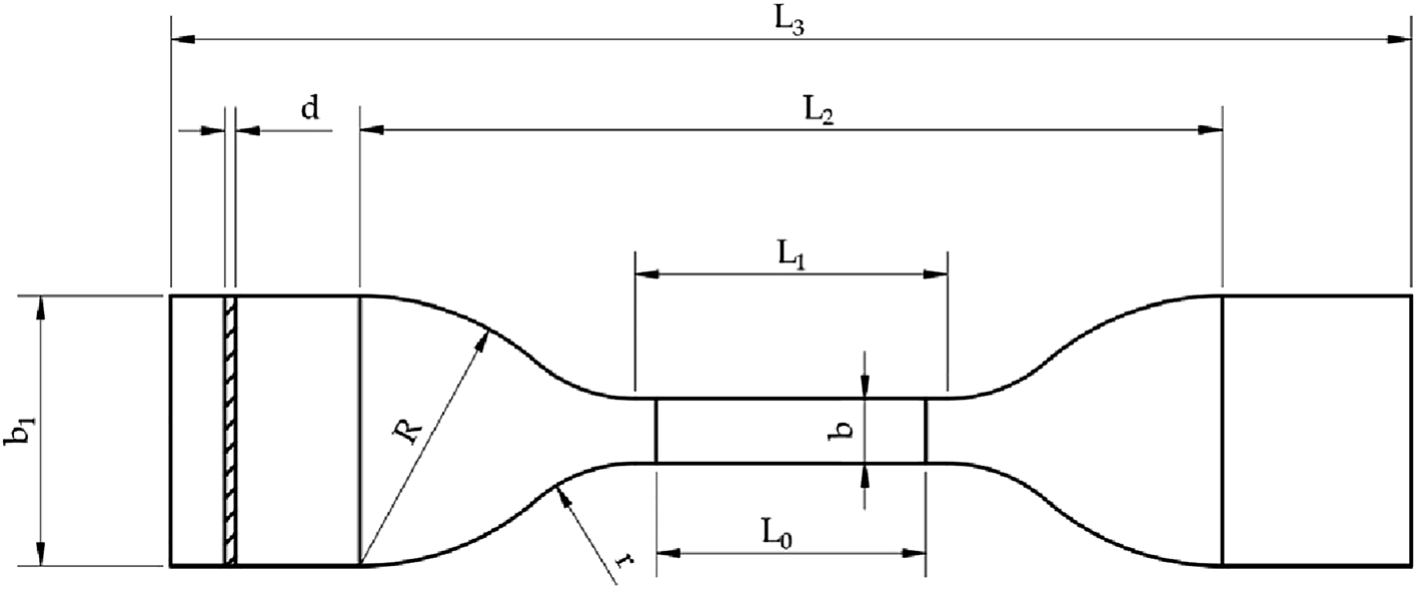
Structure and size of drip irrigation belts for tensile strength measurement: L_3_ is the total length, 115 mm; L_2_ is the initial distance between two clamps, 80 mm; L_1_ size is 33 mm; L_0_ is the distance between two markings, 25 mm; R is the large arc radius, 25 mm; r is the small arc radius, 14 mm; b is the width of the sample at that position, 6 mm; b_1_ is the width of the sample end, 25 mm; d is the thickness of the sample, 0.2 mm.

The WD-D3-7 microcomputer controlled electronic universal testing machine produced by Shanghai Zhuoji Instrument and Equipment Co., Ltd. (Shanghai, China) was adopted as the mechanical testing equipment, with a testing force range of 2 kN. The errors of the force sensor and displacement sensor of the equipment were both less than ± 0.5%. Labyrinth drip irrigation belts used in Xinjiang is thermoplastic plastic, and the wall thickness is far less than 1 mm, so the national standard GB/T13022-1991 Test Method for Tensile Properties of Plastic Film was adopted for the production and experiment of drip irrigation belts samples. The fixture clamping length was a half of L_3_-L_2_. The specific structure and size of tensile strength specimen were shown in Fig. 2.

Equation (1)^31^was used to calculate the tensile strength and maximum tension force. When each group of samples were stretched, the stretching speed was set to 10 mm/min, and the temperature in the laboratory was about 27 °C. The tensile test was repeated for 5 times for each group of samples, and the results were averaged.

1 $$\left\{ {\begin{array}{*{20}l} {\sigma_{{\text{t}}} = \frac{{\text{p}}}{{{\text{bd}}}}} \hfill \\ {F_{\max } = \sigma_{{\text{t}}} \cdot 2{\text{d}} \cdot 16} \hfill \\ \end{array} } \right.$$

where, σ_t_ is the tensile strength, MPa; p is the maximum load, N; b is the width of the specimen, mm; d is the thickness of the specimen, mm; F_max_ is the maximum tensile force of the sample, N.

#### Tests of elongation at break and natural rebound rate of drip irrigation belts

In order to investigate the change rule of plastic properties and toughness of different labyrinth drip belts under load, the elongation at break and natural rebound rate were measured. Equation (2) was used to calculate the elongation at break and natural rebound rate.

2 $$\left\{ {\begin{array}{*{20}l} {\delta_{{\text{t}}} = \frac{{{\text{G - G}}_{0} }}{{{\text{G}}_{{0}} }} \times 100\% } \hfill \\ {\delta {}_{r} = \frac{{{\text{G - G}}_{r} }}{{{\text{G}}_{{0}} }} \times 100\% } \hfill \\ \end{array} } \right.$$

where, δ_t_ is the elongation at break,%; G is the maximum distance between the markings after tensile fracture of the specimen, mm; G_0_ is the distance between the original markings of the sample, mm; δ_r_ is the natural rebound rate,%; G_r_ is the distance between the markings after unloading the sample for 30 min, mm.

#### Static load tensile tests of drip irrigation belt samples

**Figure 3:**
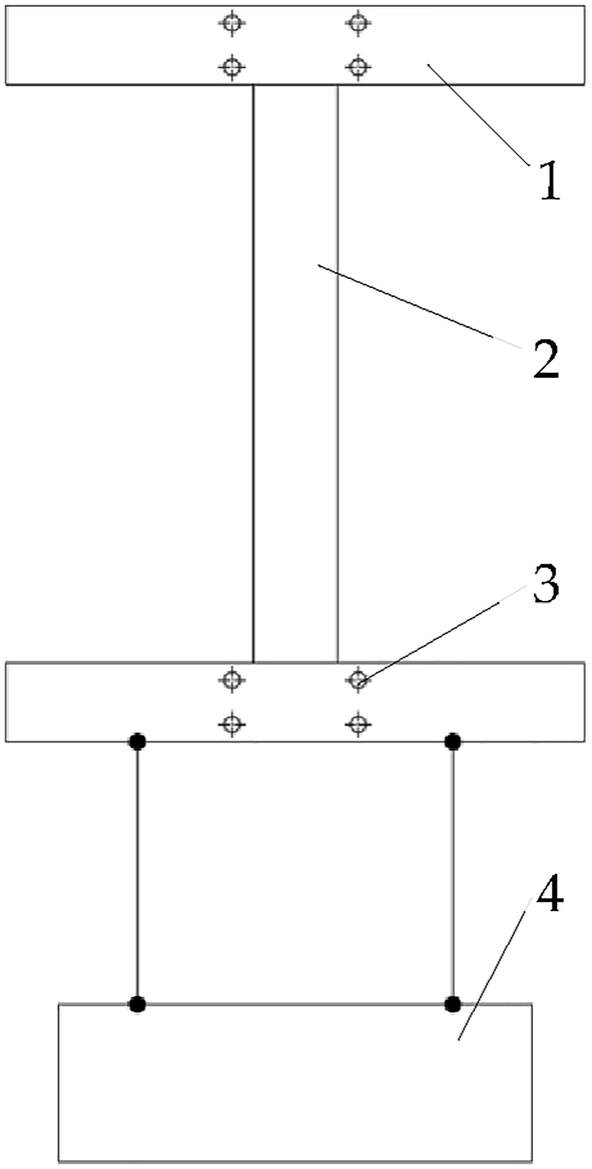
Schematic diagram of static load tensile test of drip irrigation belt samples: 1, clamp; 2, drip irrigation belt sample; 3, bolt hole; 4, 130 N weight.

According to the national standard GB/T19812.3-2008, the samples of drip irrigation belts should be able to withstand a tensile force of 130 N without fracture, and the increase of the marking distance before and after the experiment should be ≤ 5%^32^. Therefore, in this study, the static load (130 N) tensile test was carried out to analyze the change of mechanical properties of drip irrigation belt after use and its impact on mechanized recovery and cyclic utilization.The length of drip irrigation belt samples for static load tensile test was 300 mm, and 45 mm was set at both ends of the sample for clamping. Two horizontal markings were drawn on each sample with a spacing of 200 mm, the distance between each marking and the end of the clamp was 5 mm. The sample was fixed on the self-made clamping device (as shown in Fig. 3.) for experiment. During the experiment, the gravity acceleration was taken as 10 m/s^2^. That is, the gravity of a 13 kg object was taken as the 130 N static load, and the sample was slowly loaded until the 130 N weight was fully loaded on it. Then, according to the provisions of GB/T19812.1-2005, the 130 N static load was removed after 15 min, and the sample was observed whether broken or severely deformed. Finally, the sample of drip irrigation belt after the experiment was placed for 30 min, after that, the distance between the markings was measured, with the elongation relative to the original marking distance was calculated.

#### Impact strength tests of drip irrigation belt samples

In order to analyze the toughness and impact resistance of drip irrigation belt samples under impact state, impact tests were conducted on new and old drip irrigation belt samples. Drip irrigation belt belongs to soft and thin plastic with a thickness of less than 1 mm, so the pendulum method was used in this experiment. XJJ-50 simply supported beam impact tester produced by Chengde Donglai Testing Instrument Co., Ltd. (Chengde, China) was selected for this experiment. The unnotched impact strength tests was implemented at room temperature (about 27 °C). The length, width and thickness of the sample were 80 × 10 × 0.2 mm. The experiment of each group of samples were repeated 5 times, and the average was taken as the final results. The impact strength of the samples was calculated by Eq. (3).

3 $${\text{A}} = \frac{{\text{E}}}{{{\text{b}} \times {\text{d}}}} \times 10^{3}$$

where, A is the impact strength of the sample, kJ/m^2^; E is the impact energy absorbed by the sample, J; b is the width of the sample, mm; d is the sample thickness, mm.

### Chemical composition tests of drip irrigation belt samples

#### X-ray energy spectrum tests on the surface of drip irrigation belt samples

When conducting the X-ray energy spectrum experiment on the surface of the drip irrigation belt, the clean drip irrigation belt was cut into a square sample with a width and thickness of 5 × 0.2 mm. Then, the sample surface was sputter coated with ion sputter by the 108auto ion sputtering instrument (Waterford, Britain) produced by CRESSINGTON. ESCALAB 250Xi X-ray photoelectron spectrometer of Thermo Fisher Scientific Company (Waltham, USA) was used to measure the element composition on the surface of different drip irrigation belt samples. Finally, the aging degree of old drip irrigation belts was analyzed according to the results of X-ray energy spectrum experiment.

#### Infrared spectrum tests of drip irrigation belt samples

The Frontier infrared spectrometer of Perkin Elmer Company(Waltham, USA) was used in this experiment; the wavelength range of the spectrometer is 8300−350 cm^−1^, the spectral resolution is better than 0.4 cm^−1^ and the wave number accuracy is better than 0.008 cm^−1^. When the infrared spectrometer is used to detect the components of the sample of drip irrigation belt, there are three measurement modes: reflection, transmission and attenuated total reflection (ATR). However, if the reflection mode is used for the experiment, because the surface of the sample of the drip irrigation belt is rough and the color is black, the reflectivity is low, and the signal of the infrared spectrogram collected is weak; so, it is difficult to analyze the spectrogram according to the characteristic absorption peak. If the conventional transmission method is used to conduct qualitative test on the samples of drip irrigation belt, the samples need to be destroyed, and the in-situ detection cannot be achieved, and the transmission mode detection process is also cumbersome. But, ATR mode is a way of testing through surface contact, so this mode is not affected by sample thickness, surface morphology and substrate noise. In addition, ATR mode can detect samples directly and in situ, which can effectively reduce sample pretreatment. The spectrum of drip irrigation belt obtained under ATR mode is of high quality, so ATR mode was used in this study to detect the infrared spectrum of drip irrigation belt samples.

### Statistical analysis

The mean and standard deviation were calculated for each group of the test results about mechanical properties and X-ray energy spectrum data of drip irrigation belt samples. Normality test of the experimental data was made by the way of Shapiro–Wilk, and the homogeneity test of variance was performed by Levene’s Test. Then, one-way ANOVA was separately performed to test the significance of the difference of the population means about the experimental data of tensile tests, as well as that of X-ray energy spectrum tests. After that, Tukey Test was carried out for the means comparisons of above relative tests’ data to analyse the significance of the difference between the results of each group of drip irrigation belt samples. Tukey test is one of the widely used methods for multiple comparisons. An important advantage of the Tukey test is that it is very simple and does not require complex statistical knowledge. In addition, the required experimental samples are also relatively small. Besides, in order to study the correlation of oxygen content and the corresponding tensile strength of drip irrigation belt samples, the regression analysis was performed.

*Statement* All the methods included in the study are in accordance with the relevant institutional, national, and international guidelines and legislation^33^.

## Results

### Test results of mechanical properties of drip irrigation belt samples

#### Test results of tensile strength of drip irrigation belt samples

**Figure 4:**
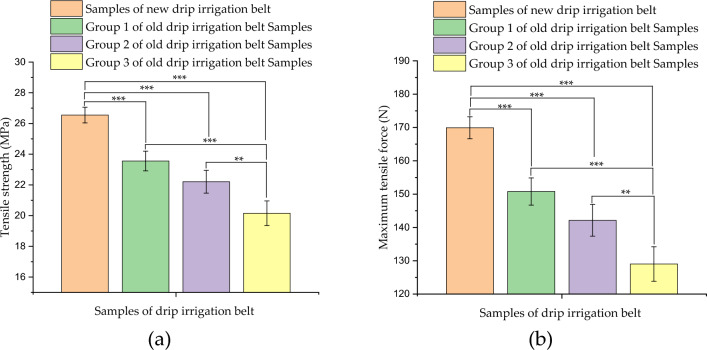
Tensile test results of drip irrigation belt samples: (**a**) tensile strength test results; (**b**) maximum tensile force of drip irrigation belts; ** represents very significant (*P* < 0.01), and *** represents extremely significant (*P* < 0.001).

The tensile strength and corresponding maximum tensile load test results of drip irrigation belt samples were shown in Fig. 4. The minimum and average tensile strength of the new drip irrigation belt samples were 25.97 MPa and 26.55 MPa respectively. While the minimum and average tensile strength of the group 1 old drip irrigation belt samples were 22.68 MPa and 23.56 MPa, respectively; the minimum and average tensile strength of the group 2 old drip irrigation belt samples were 20.96 MPa and 22.21 MPa, respectively; the minimum and average tensile strength of the group 3 old drip irrigation belt samples were 18.96 MPa and 20.16 MPa, respectively. It can be seen from the above experimental data that the average tensile strength of group 1, group 2 and group 3 of the old drip irrigation belt samples was 11.26%, 16.34% and 24.06% lower than that of the new drip irrigation belt samples respectively; the average values of the maximum tensile force of the old drip irrigation belt samples in group 1, group 2 and group 3 were 150.78 N, 142.14 N and 129.02 N respectively.

Furthermore, normality test results showed that at the 0.05 level, each group of experimental data was significantly drawn from a normally distributed population. The Levene’s Test for homogeneity of variance showed that at the 0.05 level, the population variances were not significantly different. Based on the above test results, one way ANOVA was performed and the results showed that at the 0.05 level, the population means were significantly different. Then, the results of Tukey Test for the means comparisons of tensile tests of drip irrigation belt samples were shown in the Fig. 4a,b. The tensile strength of the old drip belt samples in each group was significantly different from that of the new drip belt samples. Moreover, among the three groups of old drip belt samples, the tensile strength of old drip belt samples in group 1 and group 3 had an extremely significant difference; the tensile strength of the old drip belt samples in group 2 and group 3 was very significantly different.

#### Determination results of elongation at break and natural rebound rate

**Figure 5:**
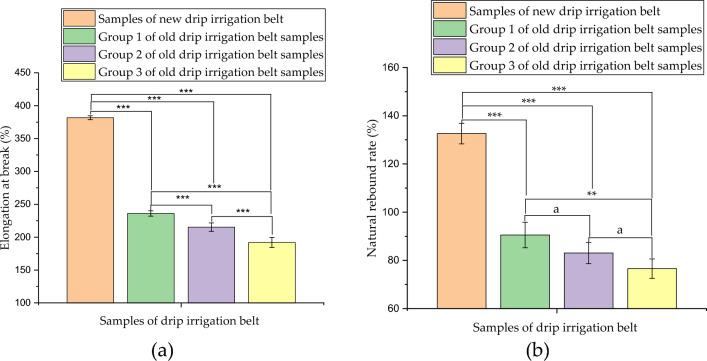
Measurement results of elongation at break and natural rebound rate of drip belt samples: (**a**) elongation at break; (**b**) natural rebound rate; a represents not significant (*P* > 0.05), ** represents very significant (*P* < 0.01), and *** represents extremely significant (*P* < 0.001).

The measurement results of elongation at break and natural rebound rate of drip irrigation belt samples were shown in Fig. 5. According to the measurement results of elongation at break (Fig. 5a), the elongation at break of the new drip irrigation belt samples was 381.67%; while the elongation at break of the group 1 old drip belt samples was 236.06%, which was 38.15% lower than that of the new drip belt samples; the elongation at break of the group 2 old drip belt samples was 215.26%, which was 43.60% lower than that of the new drip belt samples; the elongation at break of the group 3 old drip belt samples was 191.92%, which was 49.71% lower than that of the new drip belt samples. It can be seen from the natural rebound rate measurement results (Fig. 5b) that the natural rebound rate of the new drip irrigation belt samples was 132.59%, while that of the group 1, group 2 and group 3 of old drip irrigation belt samples decreased to 90.56%, 83.04% and 76.59%, respectively. In addition, one way ANOVA was conducted on the results of elongation at break and natural rebound rate tests. One way ANOVA results showed that at the 0.05 level, the population means of both elongation at break and natural rebound rate were significantly different. The specific one way ANOVA results of elongation at break and natural rebound rate were shown in the Fig. 5a,b, respectively.

#### Test results of elongation and impact strength of samples

The static load elongation and impact strength test results of each group of drip irrigation belt samples were shown in Table 1. The results of static load tensile tests showed that the samples of each group of drip irrigation belt did not break under 130 N tension, and the change of the marking distance of the samples before and after the test was less than 5%. However, the plastic deformation of the old drip irrigation belt samples was relatively large. The maximum elongation of the new drip irrigation belt samples was 2.2%, and the average was 2.1%. However, the maximum elongation of the group 1 old drip irrigation belt samples reached 3.3%, with an average of 3.2%; the group 2 of the old drip irrigation belt samples reached 3.9%, with an average of 3.7%; the group 3 of the old drip irrigation belt samples reached 4.3%, with an average of 4.1%. Therefore, the elongation of the old drip belt samples under static load was far greater than that of the new drip belt samples; especially that of the group 3 old drip belt samples was close to 5%. One way ANOVA results showed that at the 0.05 level, the population means of elongation were significantly different. The results of Tukey Test for the means comparisons of elongation tests of drip irrigation belt samples showed that the elongation of the old drip belt samples in each group was extremely significantly different (*P* < 0.001) from that of the new ones. Additionally, there were also significant differences between each group of the old drip belt samples about the elongation.

**Table 1:** Static load tensile and impact strength test results of drip irrigation belt samples.

Results	New drip irrigation belt samples		Group 1 of old drip irrigation belt samples		Group 2 of old drip irrigation belt samples		Group 3 of old drip irrigation belt samples	
	Elongation (%)	Impact strength (MPa)	Elongation (%)	Impact strength (MPa)	Elongation (%)	Impact strength (MPa)	Elongation (%)	Impact strength (MPa)
Test 1	2.0	14.2	3.3	11.4	3.7	10.9	4.2	10.2
Test 2	2.2	14.1	3.1	11.8	3.5	10.6	4.0	9.3
Test 3	2.0	13.4	3.3	11.3	3.9	10.3	3.9	9.6
Test 4	2.1	13.6	3.0	10.9	3.6	10.5	4.1	8.9
Test 5	2.2	13.9	3.2	10.7	3.8	9.9	4.3	8.7
Average value	2.1	13.8	3.2	11.2	3.7	10.4	4.1	9.3
Standard deviation	0.08	0.30	0.12	0.38	0.14	0.33	0.14	0.53

It can be seen from the impact strength experiment results that the impact strength of the old drip irrigation belt was also significantly reduced compared with that of the new drip irrigation belt. The minimum impact strength of the new drip irrigation belt samples was 13.4 MPa, and the average value was 13.8 MPa. But, in the impact strength test results of the old drip irrigation belt samples in each group, the minimum value was not more than 10.7 MPa, and the average value was not more than 11.2 MPa; In particular, the minimum and average impact strength of the group 3 old drip irrigation belt samples were only 8.7 MPa and 9.3 MPa, respectively, only 64.9% and 67.39% of the corresponding values of the new drip irrigation belt samples. One way ANOVA results showed that at the 0.05 level, the population means of impact strength were significantly different. The results of Tukey Test for the means comparisons showed that the impact strength of the old drip belt samples in each group was extremely significantly different (*P* < 0.001) from that of the new ones. Except for group 1 and 2, the impact strength of the old drip belt samples in each group was also significantly different from that of each other.

### Test results of chemical composition change of drip irrigation belt samples

#### X-ray energy spectrum test results on the surface of drip irrigation belt samples

**Figure 6:**
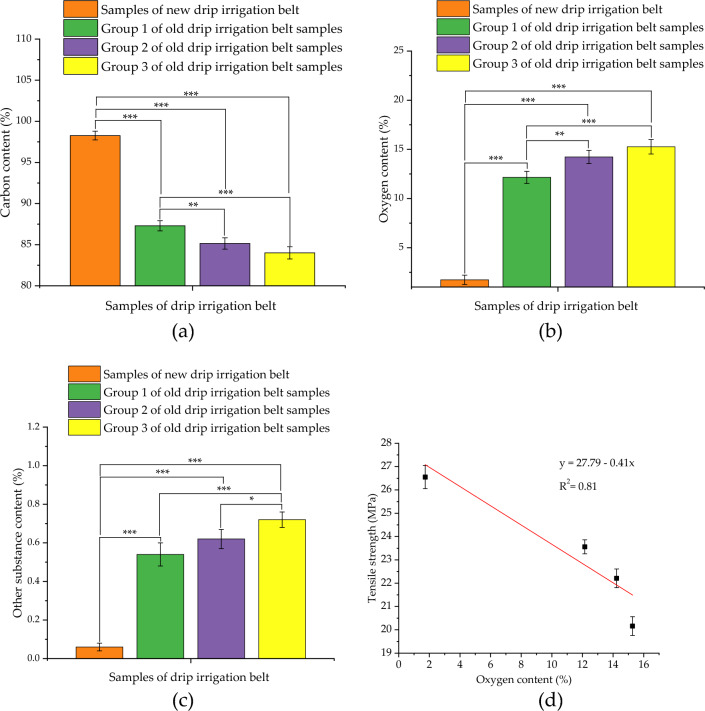
Experimental results of X-ray energy spectrum on the surface of drip irrigation belt samples: (**a**) carbon content; (**b**) oxygen content; (**c**) the content of other substances; (**d**) linear regression analysis results of tensile strength and oxygen content of drip irrigation belt samples; * represents significant (*P* < 0.05), ** represents very significant (*P* < 0.01), and *** represents extremely significant (*P* < 0.001).

The quantitative determination results of X-ray energy spectrum on the surface of new and old drip irrigation belt samples were shown in Fig. 6. It can be seen from the carbon content measurement results (Fig. 6a) that the average carbon content of the new drip belt samples, the group 1, group 2 and group 3 of old drip belt samples were 98.27%, 87.30%, 85.15% and 84.01% respectively. As shown in Fig. 6b, the average oxygen content of new drip belt samples, the group 1, group 2 and group 3 of old drip belt samples were 1.73%, 12.15%, 14.23% and 15.27%, respectively; the oxygen content percentage of the old drip irrigation belt samples in each group was much higher than that of the new drip irrigation belt samples. Besides, according to the content determination results of other substances in the drip belt samples (Fig. 6c), the percentage of other components in the new drip belt samples, the group 1, group 2 and group 3 of old drip belt samples were 0.06%, 0.54%, 0.62% and 0.72% respectively. Na, Si, Cl, Ca and Mg were detected in the samples of old drip irrigation belt in each group.

Furthermore, Normality test results showed that at the 0.05 level, each group of experimental data was significantly drawn from a normally distributed population; and the Levene’s Test for homogeneity of variance showed that at the 0.05 level, the population variances were not significantly different. Based on the above test results, one way ANOVA was performed and the results showed that at the 0.05 level, the population means were significantly different. Then, Tukey Test was performed for the means comparisons and the results were shown in the Fig. 6a,b and c. In order to explore the relationship between the oxygen content and the mechanical properties of the drip irrigation belt samples, the correlation analysis was conducted on the oxygen content percentage and the tensile strength of the drip irrigation belt samples.

The one-dimensional linear regression analysis results of the tensile strength and the oxygen content of the drip irrigation belt samples were shown in Fig. 6d. The linear fitting result was shown in Eq. (4), and its determination coefficient (R^2^) was 0.88, indicating that there was a high linear relationship between the tensile mechanical properties and the oxygen content of drip irrigation belt samples.

4 $$\left\{ {\begin{array}{*{20}l} {y = 27.79 - 0.41x} \hfill \\ {R^{2} = 0.81} \hfill \\ \end{array} } \right.$$

where, *y* is the tensile strength, MPa; *x* is the oxygen content, %; R^2^ is the determination coefficient.

#### Infrared spectrum test results of drip irrigation belt samples

**Figure 7:**
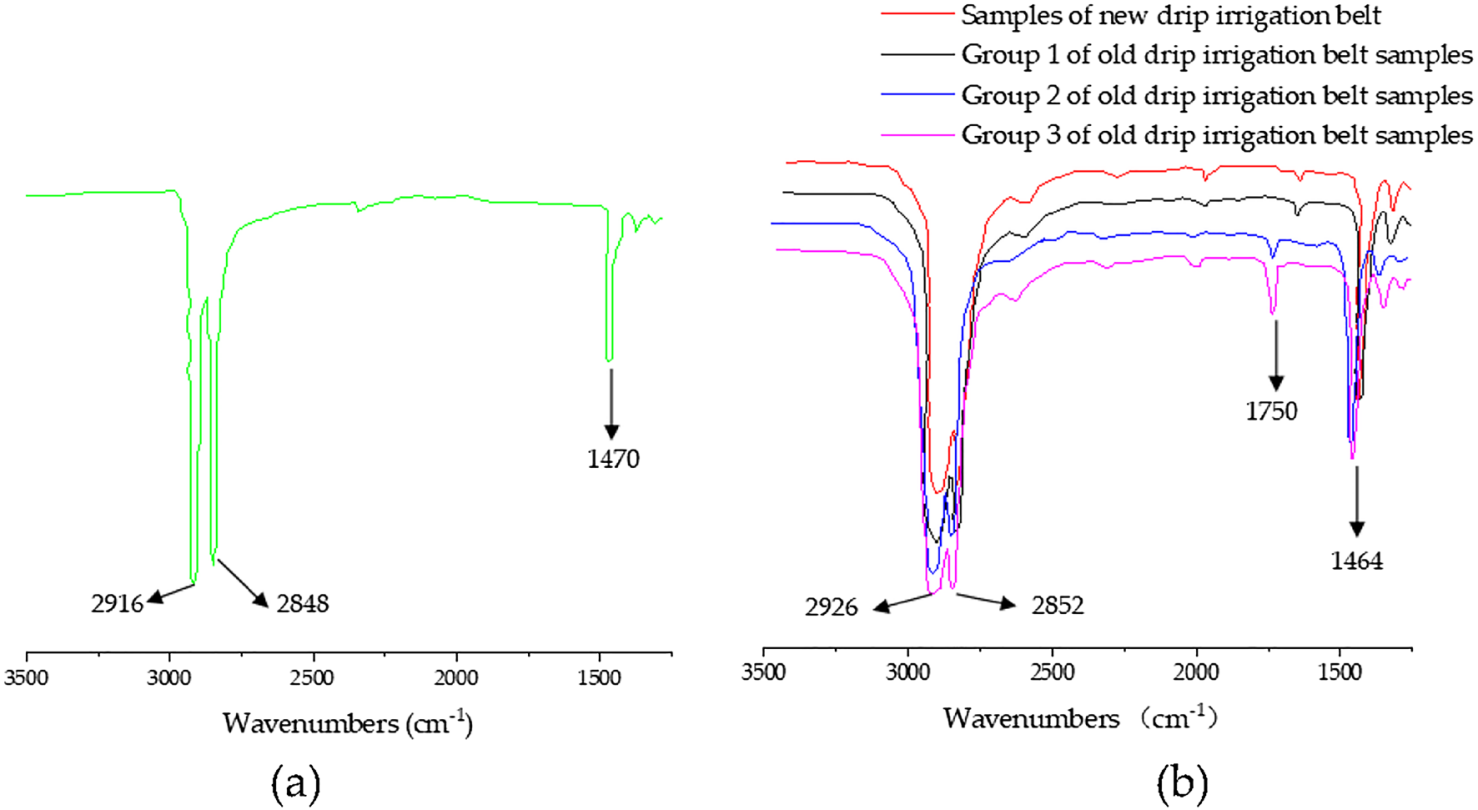
Infrared spectrum test results of pure polyethylene materials and drip irrigation tape samples: (**a**) infrared spectrum of pure polyethylene materials; (**b**) infrared spectrum of drip irrigation belt samples.

Pure PE material is composed of methylene repeating units (structural formula –CH2–CH2–), and its infrared spectrum was shown in Fig. 7a. The absorption peaks at 2916 and 2848 cm^−1^ are respectively the asymmetric stretching vibration absorption peak and symmetric stretching vibration absorption peak of methylene, and 1470 cm^−1^ is the bending vibration absorption peak of methylene. Figure 7b showed the infrared spectra of four groups of polyethylene drip irrigation belt samples. All of them had the asymmetric stretching vibration absorption peak of methylene at the wave number of 2926 cm^−1^, the symmetrical stretching vibration absorption peaks of methylene at the wave number of 2852 cm^−1^, and a flexural vibration absorption peak of methylene, at the wave number of 1464 cm^−1^.

Moreover, the infrared spectra of the four groups of drip irrigation belt samples in Fig. 7b were obviously different from those of pure polyethylene materials in Fig. 7a. In Fig. 7b, it was found that there was a carbonyl stretching vibration peak near 1750 cm^−1^ for each group of drip irrigation belt samples. In addition, it can be seen that the carbonyl stretching vibration peaks of the new drip irrigation belt samples, the group 1, group 2 and group 3 of old drip belt samples moved obviously to the high wavenumber respectively, and the spectral peak intensity also gradually became stronger. The characteristics of the carbonyl stretching vibration peak above indicated that the old drip irrigation belt samples had experienced more obvious aging than that of the new ones.

## Discussion

### The effects of aging on the mechanical properties of drip irrigation belts

The mechanical test results showed that the mechanical parameters of the old drip irrigation belt samples were significantly smaller than the corresponding mechanical parameters of the new drip belt samples. The tensile strength of the old drip irrigation belt samples was significantly reduced, which is an important reason for the mechanical recovery of drip irrigation belts in cotton fields to easily lead to belt breakage, this increases the difficulty of mechanical recovery of drip irrigation belts. During the recovery process, the tensile force of the machine on the drip irrigation belt is one of the important factors affecting the recovery efficiency and quality. In order to reduce the fracture of the drip irrigation belt, the designed load of the recovery machine on the drip irrigation belt must be less than the tensile strength of the old drip irrigation belt. For example, the maximum tensile force of the old drip irrigation belt measured in this experiment was 129.02N ~ 150.78 N, so the tensile force of the recovery machine to the drip irrigation belt should be far less than 129.02 N. The research showed that the fracture frequency of drip irrigation belt is different when different drip irrigation belt recovery machines operate^34,35^. Therefore, in the research and development of the drip belt recovery machine, we should focus on the research of the recovery technology that has small force on the drip belt and can effectively avoid the entanglement of cotton poles and other debris on the drip belt.

In addition, it is also very important to select the appropriate field recovery period of drip irrigation belts for the efficiency and quality of recovery operations. At present, most cotton farmers do not recycle drip irrigation belts until the cotton is picked, which will prolong the exposure time of drip irrigation belts in cotton fields and aggravate their aging degree. Moreover, after cotton picking, the moisture content of the cotton pole decreases and the hardness increases. When the drip irrigation belt is recycled, the cotton pole branches and the hard and sharp cotton boll shells are easy to hang the drip irrigation belt, causing a series of problems such as breaking and tearing. Therefore, the recovery of drip irrigation belt can be completed in time after the last drip irrigation in cotton field.

Furthermore, at present, the basic standards for direct reuse of old drip irrigation belts in China are that the tensile strength of old drip irrigation belts can not be lower than 19 MPa, and the elongation at break can not be lower than 270%^36^. However, from the research results, it can be seen that the minimum tensile strength of group 1, group 2 and group 3 of the old drip irrigation belt samples were 22.68 MPa, 20.96 MPa and 18.96 MPa, respectively; then, the elongation at break of the group 1, group 2 and group 3 of old drip belt samples were 236.06%, 215.26% and 191.92%, respectively. That is to say, these old drip irrigation belts cannot be directly reused.

### Changes in chemical composition of drip irrigation belts after aging

Through the experimental results of X-ray energy spectrum on the surface of drip irrigation belt, it was found that the oxygen content rate of the old drip irrigation belt samples was significantly higher than that of the new drip irrigation belt samples. The correlation analysis between the oxygen content of drip irrigation belt and the tensile strength showed that there was a significant linear negative regression relationship between them. In addition, the infrared spectrum test results showed that the carbonyl stretching vibration peak intensity of the old drip belt samples was significantly different from that of the new drip belt samples, specifically, the more severely oxidized the drip belt was, the higher the carbonyl stretching vibration peak intensity was. Furthermore, this study found that the internal chemical structure change of drip irrigation belt was closely related to the external mechanical characteristics. Therefore, infrared spectroscopy can be used to quickly detect the aging degree of drip irrigation belts; then, the mechanical parameters of drip irrigation belts can be indirectly predicted by establishing relevant mathematical models. It is of great significance to explore an efficient and accurate method for aging detection and aging degree evaluation of drip irrigation belts^37^. In addition, it is worth noting that ultraviolet radiation is the main factor affecting the aging of drip irrigation belts in cotton fields^14,15^. Therefore, in the research and development of drip irrigation belts, it is of great significance to explore suitable anti ultraviolet materials and apply them to the outer surface of drip irrigation belts to improve the anti-aging ability of drip irrigation belts^38–40^.

Besides, after aging, 0.54 ~ 0.72% Na, Si, Cl and other impurities were found in the samples of the old drip irrigation belt; this directly increases the difficulty of efficient utilization of old materials for drip irrigation belts. According to the current treatment method, the old drip irrigation belt is directly granulated after simple physical cleaning, and various polyethylene products are manufactured^41^. So, this will inevitably affect the quality and service life of the product^42,43^. Therefore, before reusing the old materials of drip irrigation belts, it is necessary to clean them based on various physical and chemical methods to remove various impurities to the maximum extent, so that the parameters of the old materials can meet the requirements of secondary use. Then, the old materials can be used to manufacture drip irrigation belts or other related products^44^. This will be conducive to the efficient utilization of the old drip irrigation belt materials. Consequently, the high efficiency cleaning and impurity removal methods based on physics and chemistry can be deeply studied to improve the physical and chemical performance indexes of the old drip irrigation belts to the maximum extent while removing impurities. Furthermore, the aging degree of different old drip irrigation belt samples was also different. Which was mainly related to a series of environmental factors such as different soil and the growth status of cotton plants where the drip irrigation belts were located.

## Conclusions

In this paper, the aging characteristics of the old drip irrigation belts were studied through experiments on the macro mechanical properties and the micro chemical composition of the drip irrigation belt samples, the differences between the new and the old drip irrigation belt samples were compared and analyzed comprehensively. The research results indicated that there were significant differences in mechanical properties and chemical components between the old drip irrigation belt and the new one in cotton fields in Xinjiang region. The mechanical properties of old drip irrigation belts significantly deteriorated after aging, which brought adverse effects on their mechanized recovery and recycling. In order to reduce the fracture rate of old drip irrigation belts during the recovery process, it is necessary to consider reducing the mechanical tensile force as much as possible when designing the old drip irrigation belt recovery machine, so that it is not greater than the fracture strength of the old drip irrigation belt. In addition, research has shown that the aging degree of old drip irrigation belts was related to their oxygen content, and the oxygen content of drip irrigation belts was also closely related to their mechanical properties. Therefore, it is expected that in the future, rapid detection of chemical components will be used to determine the degree of aging and mechanical properties parameters of drip irrigation belts. Yet, the relationship between the chemical composition change of drip irrigation belt and its aging degree and mechanical properties, were not systematically and quantitatively studied and analyzed in this study. So, in the future, it is necessary to make a profound study in this area. This will be very conducive to the rapid detection of the aging of drip irrigation belts and the recycling of old drip irrigation belts.

## Data Availability

The data presented in this study are available on request from the corresponding author.

## References

[1] Wang H, Cao H, Jiang F, Wang X, Gao Y (2022). Analysis of soil moisture, temperature, and salinity in cotton field under non-mulched drip irrigation in south Xinjiang. Agriculture.

[2] Wang X, Wang H, Si Z, Gao Y, Duan A (2020). Modelling responses of cotton growth and yield to pre-planting soil moisture with the CROPGRO-cotton model for a mulched drip irrigation system in the Tarim Basin. Agric. Water Manag..

[3] Okasha AM, Deraz N, Elmetwalli AH, Elsayed S, Falah MW, Farooque AA, Yaseen ZM (2022). Effects of irrigation method and water flow rate on irrigation performance, soil salinity, yield, and water productivity of Cauliflflower. Agriculture.

[4] Yang P, Hu H, Tian F, Zhang Z, Dai C (2016). Crop coefficient for cotton under plastic mulch and drip irrigation based on eddy covariance observation in an arid area of northwestern China. Agric. Water Manag..

[5] Liu X (2022). Analysis of crop sustainability production potential in northwest China: Water resources perspective. Agriculture.

[6] Wu N, Yang C, Luo Y, Sun L (2018). Estimating evapotranspiration and its components in cotton fields under different irrigation conditions. Pol. J. Environ. Stud..

[7] Lei Z, Zhang X, Gong J, Kamran P (2021). Influence of drip tape with labyrinth on one side on thermal oxidation aging resistance. China Plast..

[8] Rowe RK, Rimal S (2008). Depletion of antioxidants from an HDPE geomembrane in a composite liner. ASCE J. Geotechnical. Geoenviron. Eng..

[9] Achilias DS, Roupakias C, MegalokonomosLappas PA, Antonakou EV (2007). Chemical recycling of plastic wastes made from polyethylene (LDPE and HDPE) and polypropylene (PP). J. Hazard. Mater..

[10] Moly KA, Bhagawan SS, Thomas S (2002). Melt elasticity behaviour and extrudate characteristics of LLDPE/EVA blends: effect of blend ratio, compatibilisation and dynamic cross-linking. Mater. Lett..

[11] Xiong QL, Huang XY, Chen D (2013). Technology and research progress on recycling use of waste plastic. Eng. Plast. Appl..

[12] Wang Z, Li H, Zhu B, Chen C (2021). Recycling and applications of waste plastic films. China Plast..

[13] Chen X (2011). Research progress of WPC extrusion molding. Appl. Chem. Ind..

[14] Yang HM, Hu B, Wu P (2011). Stabilization effect of antioxidants and light stabilizers on UV-light aging of PE-LLD. China Plast..

[15] Zhao WL, Zhang QS, Wu ZH (2011). The relationship between light shielding agent and the resistance to light aging of LLDPE being filled. Plast. Addit..

[16] Zaharescu T, Jipa S, Henderson D, Kappel W, Maris DA, Maris M (2010). Thermal and radiation resistance of stabilized LDPE. Radiat. Phys. Chem..

[17] Carrasco F, Surina J, Colom X (1996). FTIR and DSC study of HDPE structural changes and mechanical properties variation when exposed to weathering aging during Canadian winter. J. Appl. Polym. Sci..

[18] Yang R, Liu Y, Yu J, Wang K (2006). Thermal oxidation products and kinetics of polyethylene composites. Polym. Degrad. Stab..

[19] Wong WK, Hsuan GY (2014). Interaction of an antioxidants with carbon black in polyethylene using oxidative induction time methods. Geotext. Geomembr..

[20] Tochacek J, Vratnickova Z (2014). Polymer life-time prediction: The role of temperature in UV accelerated ageing of polypropylene and its copolymer. Polym. Test.

[21] Pagès P, Carrasco F, Surina F (1996). FTIR and DSC study of HDPE structural changes and mechanical properties variation when exposed to weathering aging during canadian winter. J. Appl. Polym. Sci..

[22] Liu YP, Li H, Wei XL (2007). Study on the mechanical property of the nature aging HDPE specimens. Shandong Chem. Ind..

[23] Guo JJ, Yan H, Dai J, Hu ZD, Yang JJ (2015). Degradation behavior and oxidation products analysis for high density polyethylene during lasa tibet natural aging. J. Funct. Mater..

[24] Liu L, Huang ZY, Li YL, Ren XC (2014). The study on cracking behavior of the stress-light oxidative aging of high-density polyethylene. China Plast. Ind..

[25] Mazaher AN, Rouhallah FN, Hamidreza S, Hamid RV, Kaveh OAA (2023). Effect of different managements with drip irrigation (tape). Appl. Water Sci..

[26] Liang J, Yang Q (2009). Mechanical properties of carbon black-filled high density polyethylene antistatic composites. Reinf. Plast. Compos..

[27] Liang J (2000). Elastic behavior of LDPE/HDPE blend melts in capillary extrusion. Appl. Polym. Sci..

[28] Liang J (2009). Mechanical properties of PPS/PC/GF/Nano-CaCO3 hybrid composites. Polym. Plast. Technol. Eng..

[29] Fang J, Wan X (2008). XPS analysis of tea plant leaf and root surface. Spectrosc. Spectr. Anal..

[30] Zhou X, Chen L, Huang S, Su G, Yu Y (2014). Performance of bamboo flour/polypropylene foamed composite under accelerated weathering. Trans. CSAE.

[31] Ma JX, Lei ZK (2014). Brief analysis on product quality of drip irrigation tape in Xinjiang. Qual. Explor..

[32] Huang H, Zhang W, Lei ZK, Wang C, Zhu XC (2016). Analysis of ash in drip irrigation tape and its effect on tensile properties. Mod. Plast. Process. Appl..

[33] Faridul Hasan KM, Horváth PG, Kóczán Z, Bak M, Alpár T (2021). Colorful and facile in situ nanosilver coating on sisal/cotton interwoven fabrics mediated from European larch heartwood. Sci. Rep..

[34] Ding SS, Li TW, Wang JK, Hu K (2015). Analysis of power consumption of 4HS drip irrigation belt recovery machine. J. Chin. Agric. Mech..

[35] Guo WS, Wang ZY, Sun Y, Tian YT, Hu C, San YL (2018). Design of imitating artificial picking type drip irrigation belt recovery machine and simulation of virtual prototype. Xinjiang Agric. Sci..

[36] Yimiti MMTJ, Jumakadeer ASKDE, Guan L, Biekan RYZ, Ni LG (2018). Judgment method of aging degree of recycled materials in drip irrigation. Eng. Plast. Appl..

[37] Tang QF, Li Q, Wang JM, Zhang YX, Gao X (2021). Identification and analysis of microplastics by micro fourier transform infrared spectroscopy. China Plast..

[38] Yu ZS, Li YC, Wangle HX, Bai Y, Li Z (2021). Anti-UV aging performance of polycarbonate for laser direct structuring. Eng. Plast. Appl..

[39] Wu Q, Qu BY, Wu QH (2001). Surface photooxidation and photostabilization in photoinitiated crosslinking of polyethylene. Chem. J. Chin. Univ..

[40] Zhang ZY, Lu MF, Guo P, Xu YH, Zhang SJ (2022). Influence of ultraviolet absorbers on weather resistance of PBST film. China Plast..

[41] Kajaks J, Kalnins K, Zagorska A, Matvejs J (2017). Some exploitation properties of wood plastic composites based on recycled high density polyethylene and plywood production residues. Solid State Phenom..

[42] Miguel ML, Gonzalo MB, René SD, Osman G (2020). Recycling polypropylene and polyethylene wastes in production of polyester based polymer mortars. Constr. Build. Mater..

[43] Marsh R, Griffifiths AJ, Williams KP, Evans SL (2006). Degradation of recycled polyethylene film materials due to contamination encountered in the products’ life cycle. Mech. Eng. Sci..

[44] Omoyeni OP, Atuanya CU, Maddulety K, Aigbodion VS (2016). Utilization of maize husk/recycled low density polyethylene waste materials for composite board production. Res. Rev.: J. Mater. Sci..

